# Do the Leading Children's Hospitals have Quality Web Sites? A Description of Children's Hospital Web Sites

**DOI:** 10.2196/jmir.6.2.e20

**Published:** 2004-06-25

**Authors:** Terry Kind, Kathryn L Wheeler, Byanqa Robinson, Michael D Cabana

**Affiliations:** ^1^Department of PediatricsChildren's National Medical CenterGeorge Washington University School of Medicine and Health SciencesWashington DCUSA; ^2^Child Health Evaluation and Research (CHEAR) UnitDivision of General PediatricsUniversity of Michigan Health SystemAnn Arbor MIUSA

**Keywords:** World Wide Web, Internet, children's hospitals, hospitals, pediatric, health information, quality, quality indicators, health care

## Abstract

**Background:**

Although leading children's hospitals are recognized as preeminent in the provision of health care to children, the quality of their Web sites has not been described.

**Objective:**

To describe technical characteristics of the Web sites of leading children's hospitals.

**Methods:**

This is a cross-sectional descriptive infodemiology study. Two reviewers independently reviewed and analyzed the Web sites of 26 nationally prominent children's hospitals in June 2003, using objective criteria based on accessibility (based on age and language), attribution, completeness, credibility, currency, disclosure, readability, and other technical elements.

**Results:**

One-third of Web sites included content for children and adolescents. Twenty-four (92%) of the Web sites had health and disease-specific information. One-third contained only English, while two-thirds included other languages. All 26 Web sites included a disclaimer, although none had a requirement to read the disclaimer before accessing health and disease specific information. Twenty-four (92%) had search options. Although most (85%) listed a copyright date, only 10% listed the date last updated.

**Conclusions:**

This is the first study to examine the Web sites of leading children's hospitals. Although the Web sites were designed for children's hospitals, only a few sites included content for children and adolescents. Primary care physicians who refer patients to these sites should be aware that many have limited content for children, and should assess them for other limitations, such as inconsistent documentation of disclaimers or failure to show the date of the last Web site update. These Web sites are a potentially useful source of patient information. However, as the public increasingly looks to the Internet for health information, children's hospitals need to keep up with increasingly high standards and demands of health-care consumers.

## Introduction

Although leading children's hospitals are recognized as preeminent in the provision of health care to children, the quality of their Web sites has not been described. Providers may be interested in referring parents and patients to the Internet for pediatric information and may look to the leading children's hospitals as a source. In this paper we seek to describe technical and content characteristics of the Web sites of leading children's hospitals.

The World Wide Web is becoming a popular source of health information for patients [1]. A general rule for selecting an online source for health information is to "find a Web site that has a person, institution or organization in which you already have confidence" [2].

The public and medical providers recognize leading, tertiary care, teaching hospitals as credible sources of information [3]. Many of these institutions include children's hospitals. Because the leading children's hospitals in the United States are commonly held in high regard, a parent or patient might expect that they would also be reasonable sources of online health information. Our findings suggest that such academic Web sites may disappoint [4].

It is not clear if the best children's hospitals that provide high quality care also have Web sites that provide high quality access and content. Although numerous systems for rating the quality of health information on the Internet have been developed [5-8], to our knowledge, there has been no reported evaluation specifically assessing the Web sites of the leading children's hospitals. The criteria used in this study to assess quality involved the domains of accessibility, attribution, credibility, currency, and disclosure, and other Web site elements. The purpose of this paper is to describe the technical characteristics of these Web sites, in terms of quality and content, for the leading children's hospitals.

Our research questions are the following: Do children's hospitals that are considered to provide high quality care also have Web sites that provide high quality access and content? What are the technical characteristics of the Web sites of the leading children's hospitals?

## Methods

We conducted a cross-sectional descriptive infodemiology analysis of the Web sites of the prominent children's hospitals in the United States. We selected 26 children's hospitals based on the 2002 *United States News and World Report* and the 2002 *Child* magazine rankings of the leading children's hospitals.

### Sample Selection

Although there are many methods for selecting leading medical institutions and children's hospitals, medical providers and the public are influenced by the *United States News and World Report* ranking of "America's Best Hospitals" [9-11]. Another rating system, specific to pediatric hospitals, is published in *Child* magazine. For this study, we selected all 23 hospitals listed as leading children's hospitals from *US News and World Report* and all 10 leading pediatric hospitals from *Child* magazine. Together, these represent 26 distinct Web sites. The Internet addresses of these hospitals were published in the *US News* and *World Report* online. However, as these Web sites were not always specifically referring to the pediatric hospital, but rather to the parent medical center, reviewers searched for the correct address on the parent medical center's site, or by entering the hospital name into Google if a hospital was listed only in *Child* magazine (Table 1).

The *US News and World Report* list has been published and updated every year since 1990, and is the longest running annual ranking of hospital quality [10]. The list also represents a common source for parents when finding medical information on the Internet [11]. *US News and World Report* ranks hospitals in pediatrics based on reputation [12]. The "America's Best Hospitals" methodology was devised in 1993 by the National Opinion Research Center at the University of Chicago [13].

*Child* magazine has also published a list of leading children's hospitals that are full members of the National Association of Children's Hospitals and Related Institutions. *Child* first selects hospitals that received a score of at least 93 (91 in some circumstances) by the Joint Commission on Accreditation of Healthcare Organizations (JCAHO). These hospitals then complete a survey developed by *Child* advisory board members to identify the leading 10 children's hospitals [14].

Although these selected institutions are acknowledged as leaders, their Web sites are not necessarily the most popular (eg, as defined by the number of backlinks or a ranking in search engines such as Google). Our selection method assumes that people who are familiar with the non-Web reputations of these institutions may directly look up these institutions' Web sites, but they may not think critically about whether the sites are as reputable as the institutions themselves.

Two of the researchers (TK, MDC) independently reviewed each Web site using a set of objective criteria pre-determined by the authors. These included criteria in the domains of accessibility, attribution, credibility, currency, and disclosure, and other Web site elements. Specifically, we determined the presence or absence of the following: child-focused content and links for children, bilingual or multilingual content, health or disease specific information, references for medical information, posting of a "last update" and copyright date, an internal search engine, disclaimer and requirement to read it, option to make purchases or donations, and advertisements. For Web sites that included disease-specific information, we selected a basic text passage about asthma, and determined the readability using the Flesch-Kincaid Grade Level method, a commonly used computerized software program for scoring readability that is embedded in Microsoft Word [15].

Eysenbach et al have described five different types of criteria to evaluate the quality of a Web site [16]. These include technical characteristics, readability, design, accuracy, and completeness. To evaluate the Web sites, we included technical characteristics, readability, and completeness criteria. We did not include criteria based on Web site design, since previous studies have reported kappa scores of only 0.08 and 0.23 [16]. In addition, design criteria might not be valid for an analysis of these Web sites, since the pages might be designed for children. Since not all the Web sites offered disease-specific information, we did not include criteria for accuracy.

Data were abstracted from June 1, 2003 to June 30, 2003. Differences in classification were resolved by another reviewer (KLW or BR). We calculated kappa statistics for the dichotomous categories to describe the agreement in the initial classification of each of the characteristics. Simple counts and descriptive statistics are presented to describe the frequency of these characteristics on each hospital's Web site.

The hospital rankings from *US News and World Report* (n=23 hospitals) and from *Child* magazine (n=10 hospitals) are listed in Table 1, along with their Internet addresses. Combined, the two lists included a total of 26 hospitals. Seven hospitals appeared on both lists. All 26 leading hospitals in the initial sample had Web sites specific to pediatrics or to the children's hospital.

**Table 1:** Leading Hospital Web Sites Included in Analysis

Hospital	Web Site Address as Listed By US News*	Pediatric or Children's Hospital Web Site Address*
Children's Hospital Boston	childrenshospital.org	Same
Children's Hospital of Philadelphia	chop.edu	Same
Johns Hopkins Hospital	hopkinsmedicine.org	Hopkinschildrens.org
Children's Hospital, Denver	tchden.org	Same, also thechildrenshospital.org
Children's Hospital of NY Presbyterian	nyp.org	childrensnyp.org
Children's Hospital of Pittsburgh	chp.edu	Same
University Hospitals of Cleveland	uhhs.com	rainbowbabies.org
Texas Children's Hospital, Houston	txchildrens.org	Same
Children's Hospital Medical Center, Cincinnati	cincinnatichildrens.org	Same
Children's Memorial Hospital, Chicago	childrensmemorial.org	Same
Children's Hospital, Los Angeles	childrenshospitalla.org	Same
University of California, San Francisco Medical Center	ucsfhealth.org	ucsfhealth.org/childrens/index.html
UCLA (Mattel Children's Center)	healthcare.ucla.edu	peds.ucla.edu
Massachusetts General Hospital	mgh.harvard.edu	massgeneral.org/mghfc/
Lucile Packard Children's Hospital (Stanford)	stanfordhospital.org	lpch.org
Mayo Clinic	mayo.edu	mayo.edu/pediatrics-rst/
Children's National Medical Center, DC	dcchildrens.com	Same
Children's Hospital and Medical Center, Seattle	seattlechildrens.org	Same
Duke University Medical Center	dukehealth.org	dukehealth.org/health_services/childrens_health.asp
Miami Children's Hospital	mch.org	Same
Yale-New Haven Hospital	ynhh.org	ynhh.org/ynhch/ynhch.html
University of Michigan Hospitals	med.umich.edu	med.umich.edu/mott
St. Christopher's Hospital, Philadelphia	stchristophershospital.com	Same
St Louis Children's Hospital	n/a	stlouischildrens.org
Children's Mercy Hospital, Kansas City	n/a	childrens-mercy.org
Primary Children's Medical Center, Salt Lake City	n/a	ihc.com/xp/ihc/primary

^*^ all addresses in this table have URLs (Uniform Resource Locators) prefixed with http//:www(HyperText Transfer Protocol; World Wide Web). n/a = not applicable, ie, the hospital was only listed in *Child* magazine, which did not list the URL

Characteristics of the Web sites are listed in Table 2. All 26 Web sites included a disclaimer and/or privacy policy and/or terms of use. Twenty-four (92%) of the Web sites contained health and disease-specific information. None of the sites required the user to log in before reading health and disease-specific information. None of the sites included a requirement to read a disclaimer before accessing their health and disease-specific information. Twenty-four (92%) of the Web sites had search options.

We measured accessibility of the Web sites for children, based on whether or not the Web site included information for children or recommended links. Although the Web sites were designed for children's hospitals, only one-third included content for children and adolescents. Accessibility was also examined with regard to multilingual content. One-third of the Web sites contained only English, while two-thirds included other languages.

In terms of completeness, 92% provided health or disease-specific information. Two-thirds (65%) provided additional or recommended Web sites. With respect to technical features, 92% of the sites allowed the user the option of searching the site.

All the sites offered a disclaimer and/or privacy policy. Although most Web sites (85%) listed a copyright date, fewer than 10% (2 hospitals) listed the date of the last Web site update.

All Web sites provided information about making a donation to the hospital; however, only one site (4%) had advertisements for organizations or companies other than the hospital itself [4].

Kappa statistic calculations revealed that the agreement between the two reviewers exceeded expected agreement for all variables assessed. Kappa ranged from 0.24 to 1.00.

**Table 2:** Characteristics of Web Sites for the Leading Children's Hospitals

Domain:	Does the Web site have	n (%)	Κ*
Accessibility (children)	content for children (educational or non-educational games)?	10 (38.5)	0.53
Accessibility (children)	recommended links for children?	9 (34.6)	0.49
Accessibility (teens)	recommended links or content for teens?	10 (38.5)	0.65
Accessibility (language)	English language only (no other languages)?	9 (34.6)	0.24
Attribution, Completeness	recommended links or resources for more information?	17 (65.4)	0.35
Completeness	Health or disease specific info?	24 (92.3)	0.34
Credibility, Conflict of Interest	purchase or donation option?	26 (100)	1.00
Credibility, Conflict of interest	advertisements other than for hospital itself?	1 (3.9)	0.47
Currency	copyright date on main (home) page?	22 (84.6)	0.90
Currency	has date last updated on main (home) page?	2(7.7)	0.34
Disclosure	disclaimer, privacy policy, or terms of use?	26 (100)	1.00
Disclosure, Accessibility	requirement to read disclaimer prior to accessing health information?	0 (0)	n/a
Disclosure, Accessibility	requirement to log in prior to accessing health information?	0 (0)	n/a
Readability	8^th^ grade or lower readability for disease specific info (asthma)?	8 of 21 (38.1)	n/a
Technical Features	search option?	24 (92.3)	1.00
Technical Features	option to email child or join an online community?	14 (53.8)	0.45

^*^ Kappa score, reflecting the agreement between the two raters. 1.0 represents perfect agreement.

## Discussion

### Main findings

This is the first study to examine the Web sites of the leading children's hospitals. Although all the commonly recognized leading children's hospitals have their own Web sites, style and content vary. Many of the Web sites lacked information for children. We also found that access to many sites was limited by the reading level and the language(s) in which the information was offered. In addition, although many had disease-specific information, the currency of such information was not described.

Given increasing use of the Internet as a source for health information by parents and patients [1], we expected that most of the Web sites for the leading children's hospitals would include pediatric health information, especially educational content intended specifically for children. However, this study shows that although the Web sites created by hospitals are dedicated to children, only one-third have information specifically for such an audience. Internet users with children (ie, parents), and pediatric providers who refer children to these sites for educational content would be disappointed by most of the sites.

In addition to being a useful and trusted source of patient information, these sites can easily guide and potentially link the parent or patient to information about a specialist at the hospital. As a result, children's hospitals are in a unique position to provide disease-specific information on the Internet, and theoretically may be more useful to health-care consumers than government sites (eg, Centers for Disease Control and Prevention or the National Institutes of Health) or private organization sites (eg, the American Lung Association), which traditionally do not contain links to providers or centers for care. By recommending high quality Web sites, pediatricians and other providers can assist parents and patients in becoming more involved in their own care and in learning about their health [17].

However, providing disease-specific information requires that such information be updated regularly. One reason leading children's hospitals might be considered "top" is because they remain current, on the cutting edge of medical research and technology. Because Web sites can easily be updated, users probably assume that information on the Internet is up-to-date [8].

Yet as the results of this study show, fewer than 10% of the Web sites of the leading children's hospitals assessed in this study posted the date the site was last updated on their home pages. It is not clear to the Web site audience how current the information is. Out-of-date information can contribute to inaccurate patient information. For example, McClung reviewed Internet sources regarding the treatment of childhood diarrhea and found that only 20% of sites, including those of traditional medical institutions, had information consistent with the most recent American Academy of Pediatrics guidelines for the management of acute diarrhea [18]. Web sites should post the date of the most recent update.

Given ethical concerns and legal regulations about Internet usage as it relates to health-care services [19-22], it was expected that all sites would include a disclaimer, privacy policy, and/or terms of usage. Yet, while some sites prompted the user to "read this disclaimer first," none required that the user read or view the disclaimer in order to gain access to the information on the Web site. Disclaimers and terms of use contain important cautions regarding the limitations of the information on a Web site, stating, for example, that it does not substitute for a physician visit or that the Web site is intended only for physician use. It has yet to be determined whether users actually read disclaimers if not compelled to do so. If they do not read the disclaimer, Web site visitors may misuse the information and could put themselves at risk by not seeking care from a health professional. While a prior evaluation of reported cases of harm associated with the use of Internet-based health information yielded just a few reported cases of harm, this finding could be due to a true low risk, underreporting, or bias [23]. Yet, a one-time prompt on the Web site would be a reasonable way to promote reading of the disclaimer without placing an undue burden on the user.

Accessibility of the Web site was also measured in terms of language. The children's hospitals we surveyed were from different parts of the country, with different populations to serve. Although we only considered Web sites from the leading children's hospitals in the United States where English is the primary language, two-thirds of the Web sites did include languages other than English. This is a commendable effort on the part of the hospitals to reach out to their non-English-speaking patients. The differences in language availability may reflect the differences in the population of patients served by each hospital.

### Limitations

There are several limitations to this study. Although most of the Web sites in this study contain health and disease-specific information, we did not evaluate the accuracy of this information. Not all sites contained disease-specific information, and some of the sites had disease-specific content that did not differ from content on other sites, as it was purchased from a third party. Nonetheless, future investigation of disease-specific content would be necessary to evaluate on this criterion.

In addition, the low kappa scores for certain variables in this study may relate to Web site design. Because we were evaluating Web sites as opposed to specific Web pages, the range in kappas may reflect the differences in the ability to find the specific information among the different Web pages at one hospital Web site.

Another limitation of this study is that, although we evaluated the Web sites whose target audience is public, this was not a natural experiment using actual consumers of Internet-based pediatric health information. Further research can clarify how parents, for example, use the Internet for health information.

There are many criteria upon which a Web site can be evaluated. Our study did not ask whether sites had "contact us" information, which would attest to the accountability of the site. In addition, information on Web team composition would assist the user in learning who specifically authored the site. Although we did not include all the possible domains upon which a Web site can be evaluated, we chose several that are relevant to the pediatric community as well as those that are commonly employed in literature reports of Web site evaluation [5-8].

### Conclusion

This is the first study to examine the Web sites of the leading children's hospitals. Surprisingly, only one-third had links or content for either children or adolescents. All had disclaimers but none required users to read the disclaimer. Almost all of the Web sites contained health and disease-specific information, and many had multilingual information on their sites. The Web sites of the leading children's hospitals are a potentially useful source of patient information for primary care physicians to offer to their patients. However, this study indicates that the current Web sites of children's hospitals have several limitations.

This study suggests methods to improve Web sites for children's hospitals. Specifically, those responsible for such Web sites could provide educational content for children or provide quality links, as well as improve the readability levels of their content. In terms of technical features, Web sites should describe and maintain the currency of the information on their sites, and maintain appropriate disclaimers with adequate prompting of users to read such disclaimers. Finally, based on the population that the children's hospital serves, the Web site should provide reasonable multilingual options.

For providers interested in referring parents and patients to the Internet for pediatric information, this study demonstrates variability with respect to the leading children's hospital Web sites. These sites could be potential sources of additional information and patient education; however, providers should examine the extent that the Web sites they recommend meet the above quality criteria. As the public increasingly looks to the Internet for more health information, children's hospitals need to keep up with the increasing standards and demands of health-care consumers.
